# Clinical impact of antibiotic resistance in odontogenic infections: a 12-year analysis of 740 cases

**DOI:** 10.1007/s00784-025-06687-6

**Published:** 2025-12-03

**Authors:** Martin Fischer, Jürgen Rödel, Stefan Schultze-Mosgau, Konrad Tolksdorf

**Affiliations:** 1 Department of Oral and Cranio-Maxillofacial-Surgery/Plastic Surgery, Jena University Hospital, Friedrich Schiller University of Jena, Am Klinikum 1, 07747 Jena, Germany; 2 Institute of Medical Microbiology, Jena University Hospital, Friedrich Schiller University of Jena, Am Klinikum 1, 07747 Jena, Germany

**Keywords:** Odontogenic infections, Anti-bacterial agent, Drug resistance, Microbial, Clindamycin, Penicillin hypersensitivity, Retrospective studies

## Abstract

**Objectives:**

This study aimed to investigate the microbial spectrum and resistance patterns in surgically treated odontogenic infections and to assess the clinical impact of resistance, including systemic complications and hospitalization. Resistance rates were additionally evaluated in relation to reported penicillin hypersensitivity.

**Methods:**

A total of 740 inpatient cases with microbiological testing from surgically treated odontogenic infections at the Jena University Hospital from January 1, 2012, to December 31, 2023, were analyzed. Resistance rates were assessed at infection level. Time trends were analyzed using Poisson regression. Associations between resistance rates and clinical variables were evaluated using binary logistic regression.

**Results:**

Clindamycin resistance was observed in 38.9% of infections, while amoxicillin/clavulanate (6.9%) and moxifloxacin (4.5%) showed lower resistance rates. No statistically significant trends in resistance rates were observed. Moxifloxacin resistance increased the risk of systemic complications (OR: 10.875; 95%-CI: 2.364–50.017; *p* = 0.002), while no significant associations were found between antibiotic resistance and prolonged hospitalization. A history of penicillin hypersensitivity was associated with increased clindamycin resistance (OR: 2.156; 95%-CI: 1.038–4.480; *p* = 0.04).

**Conclusions:**

Clindamycin exhibits high resistance rates in odontogenic infections and should be critically re-evaluated as empirical therapy, especially in patients with penicillin hypersensitivity. Given the overall low resistance rates to moxifloxacin, resistance to this agent indicated highly resistant infections and was associated with an increased risk of systemic complications.

**Clinical relevance:**

Continuous resistance surveillance and adaptation of empirical therapy are essential for managing severe odontogenic infections and reducing life-threatening infection-related complications, emphasizing the need for antimicrobial stewardship.

**Supplementary Information:**

The online version contains supplementary material available at 10.1007/s00784-025-06687-6.

## Introduction

Odontogenic infections are among the most frequent causes of outpatient and inpatient consultations in dental, oral and maxillofacial medicine worldwide [1]. Most of these infections are of endodontic origin, typically arising from apical pulpitis with bacterial invasion of the periapical tissues [2, 3]. They usually originate from the physiological oral microbiota and are predominantly polymicrobial, involving both aerobic and anaerobic bacteria [1, 4–8]. The most frequently isolated aerobes include viridans streptococci, *Staphylococcus aureus*, and coagulase-negative staphylococci, while *Prevotella spp.*, *Bacteroides spp.*, and *Fusobacterium spp.* represent the predominant anaerobic species [5, 9–12].

The cornerstone of therapy consists of surgical incision and drainage to release pus and ventilate the affected area, accompanied by immediate or timely management of the odontogenic source. While this approach is typically sufficient for uncomplicated odontogenic infections [13, 14], severe cases frequently require adjunctive systemic antibiotic therapy to improve clinical outcomes [5]. This is particularly critical given the risk of life-threatening complications, such as sepsis [15–17], endocarditis and pericarditis [18, 19], descending mediastinitis [20, 21], cervical necrotizing fasciitis [22–24], jugular vein thrombosis [15, 25, 26], and airway-related complications including pneumonia and airway obstruction [18, 27, 28]. Given the inherent delays associated with microbial culture and susceptibility testing, empirical antibiotic therapy is often initiated before pathogen identification [7, 29]. Knowledge of the prevailing microbial spectrum is therefore essential for timely and effective antibiotic therapy and may decisively impact disease progression and clinical outcomes in severe infections [8].

However, selecting appropriate empirical antibiotic therapy for severe odontogenic infections remains challenging due to evolving antimicrobial resistance patterns [30]. These shifts are driven by evolutionary mechanisms such as genetic mutations, horizontal gene transfer, and alterations in gene expression or metabolism [4, 29, 31]. In recent years, inappropriate antibiotic use, including unnecessary prescriptions, deviations from clinical guidelines, and self-medication, has further accelerated the development of antimicrobial resistance [4, 10, 32]. In addition, the widespread empirical labeling of penicillin hypersensitivities based on nonspecific or misinterpreted symptoms often leads to the use of less effective antibiotics, thereby contributing to resistance development [33–35]. Consequently, penicillin, once the first-line agent for odontogenic infections, now increasingly fails to provide adequate coverage, requiring broader-spectrum or alternative agents [36].

Despite their clinical relevance, recommendations for empirical antibiotic therapy vary widely between countries, institutions, and clinicians, reflecting a lack of consensus and underscoring the need for standardized, evidence-based strategies [4, 13, 37]. Furthermore, current data on causative pathogens and resistance profiles in odontogenic infections remain limited and only few studies have investigated the potential impact of antimicrobial resistance on clinical outcomes in this context [38–40].

Therefore, this retrospective clinical study aims to address these gaps by systematically characterizing microbial spectra and resistance trends over time in a large, well-defined inpatient cohort with complicated odontogenic infections. It further evaluates the association between antibiotic resistance and adverse clinical outcomes, specifically systemic complications and prolonged hospitalization, and investigates the influence of reported penicillin hypersensitivity on resistance patterns. By correlating microbiological data with clinically relevant endpoints, this study seeks to provide evidence that may guide empiric treatment strategies and support antimicrobial stewardship in dentistry.

## Methods

All patients treated surgically for odontogenic infections between January 1, 2012, and December 31, 2023, at the Department of Oral and Maxillofacial Surgery, University Hospital Jena, were eligible for inclusion.

Cases were identified via the hospital’s electronic health records using SAP IS-H (SAP SE, Walldorf, Germany) software, based on ICD-10-GM diagnosis codes (Supplementary Table 1) and OPS procedure codes (Supplementary Table 2). After exclusion of outpatient cases (*n =* 744), duplicates (*n =* 130), miscoded (*n =* 4), and non-odontogenic infections (*n =* 95), 997 inpatient cases remained. Of these, 740 underwent microbiological sampling and were included in the final analysis (see Supplementary Fig. 1 for exclusion criteria).

Surgical treatment involved intraoral and/or extraoral incision and drainage procedures, with concurrent elimination of the odontogenic focus where feasible. Empirical antimicrobial therapy was administered in accordance with the German S3-guideline on odontogenic infections, using ampicillin/sulbactam or amoxicillin/clavulanate, adjusted for body weight and renal function. In cases of reported penicillin hypersensitivity, clindamycin was administered.

Microbiological specimens, either wound swabs or native tissue, were collected intraoperatively. Whenever clinically feasible, samples were obtained prior to the administration of antibiotics, based on infection severity and patient condition.

All specimens were transferred to the microbiology laboratory within two hours of surgery and cultured using standard procedures. Aerobic cultures were incubated for 48 h, with daily checks. Anaerobic cultures were incubated for 96 h.

Species identification was performed using Vitek MS (bioMérieux, Nürtingen, Germany). Antimicrobial susceptibility testing was performed using either the Vitek 2 system (bioMérieux) or the disk diffusion test. Breakpoints were interpreted according to the European Committee on Antimicrobial Susceptibility Testing (EUCAST) criteria (www.eucast.org).

To ensure validity of findings, only microbiological isolates obtained at the time of initial surgical intervention were included, thereby minimizing confounding from secondary in-hospital colonization. Resistance analyses were limited to infections for which susceptibility testing was available for the respective antibiotic. An infection was classified as resistant if at least one corresponding bacterial isolate demonstrated resistance.

Penicillin hypersensitivity status was assessed as a binary variable. Inpatient length of stay was measured in days, excluding cases where hospitalization duration was affected by non-infectious factors such as unrelated comorbidities or discharge against medical advice or in-hospital mortality. An adjusted dataset was used to establish the baseline length of stay. Prolonged hospitalization was defined as a duration exceeding one standard deviation above the mean. The primary clinical endpoints analyzed were presence of systemic complications and prolonged hospitalization, both treated as binary variables.

Statistical analysis was performed using IBM SPSS Statistics v29.0 (IBM Corp., Armonk, NY, USA). Statistical significance was set at *p <* 0.05. Descriptive statistics were used to summarize clinical and microbiological data. Resistance trends over time were analyzed using Poisson regression models. The annual number of resistant infections served as outcome variable and calendar year as predictor. To account for variations in the number of tested infections per year, the annual number of susceptibility tests per antibiotic served as an offset. Where overdispersion was present (deviance/df > 1), negative binomial regression was used. Final model selection was determined by the Akaike Information Criterion (AIC) and likelihood-ratio tests. Results included incidence rate ratios (IRR) together with their corresponding 95% confidence intervals (95%-CI). Binary logistic regression was applied to analyze antibiotic resistance in relation to clinical variables. Results are presented as odds ratios (OR) with corresponding 95%-CIs. Graphical representations were generated using GraphPad Prism 9 (GraphPad Software, San Diego, CA, USA).

## Results

Of the included patients, 418 (56.5%) were male and 322 (43.5%) female. The mean age was 48.5 ± 21.5 years (range: 2–95 years). An overview of relevant comorbidities and patient-related risk factors is provided in Supplementary Table 3. The average duration of hospitalization was 6.5 ± 5.0 days (range: 0–70 days). Variations from this duration were documented in 45 cases (6.1%) due to discharge against medical advice, in 23 cases (3.1%) due to diagnostics or treatment for unrelated conditions or complications, and in 2 cases (0.3%) due to fatal outcomes. Excluding these cases resulted in an adjusted mean hospitalization of 6.2 ± 3.3 days (range: 1–37 days).

The study cohort comprised 225 localized odontogenic infections (30.4%) and 515 maxillofacial space infections (69.6%). A detailed overview of the involved maxillofacial spaces is provided in Supplementary Table 4. Surgical management consisted of intraoral incision and drainage in 342 cases (46.2%) and extraoral incision and drainage in 398 cases (53.8%). Systemic complications were documented in 34 cases (4.6%). A comprehensive summary of these complications is presented in Supplementary Table 5. Prolonged hospitalization was observed in 57 patients (6.7%).

Overall, infections were predominantly polymicrobial, with a mean of 2.5 microbial species per infection. Monomicrobial infections were identified in 160 cases (21.6%). A total of 1,476 bacterial and 40 fungal isolates were reported in microbiological findings. These included 116 different bacterial species across 39 genera and six fungal species from three genera. Of the bacterial isolates, 796 (53.9%) were aerobic or facultative anaerobic, while 680 (46.1%) were strictly anaerobic organisms. The ten most frequently isolated aerobic and facultative anaerobic species are listed in Table 1, while the most common strictly anaerobic isolates are presented in Table 2. A list of all isolated microorganisms is provided in Supplementary Table 6.

**Table 1:** Frequency of detected aerobic and facultative anaerobic bacterial pathogens

Pathogens	Number of isolates	Percent (%)
Viridans streptococci	435	28.7
Coagulase-negative staphylococci	105	6.9
*Haemophilus spp.*	54	3.6
*Neisseria spp.*	36	2.4
β-hemolytic streptococci	33	2.2
*Eikenella corrodens*	31	2.0
*Staphylococcus aureus*	20	1.3
*Capnocytophaga spp.*	14	0.9
*Escherichia coli*	12	0.8
*Klebsiella spp.*	9	0.6

Presentation of the ten most frequently detected aerobic or facultative anaerobic bacteria from microbiological reports. Absolute frequencies and percentage distribution in relation to the total of all isolates (*n* = 1,516) are shown

**Table 2:** Frequency of detected strictly anaerobic bacterial pathogens

Pathogens	Number of isolates	Percent (%)
*Prevotella spp.*	337	22.2
Peptostreptococci	71	4.7
*Veillonella spp.*	71	4.7
*Fusobacterium spp.*	68	4.5
*Bacteroides spp.*	50	3.3
Propionibacteria	43	2.8
Peptococci	25	1.6
*Actinomyces spp.*	5	0.3
*Cutibacterium acnes*	4	0.2
*Slackia exigua*	3	0.2

Presentation of the ten most frequently detected strictly anaerobic bacteria from microbiological reports. Absolute frequencies and percentage distribution in relation to the total of all isolates (*n* = 1,516) are shown

Among the fungal isolates, *Candida spp.* predominated (*n* = 39; 2.6%), whereas *Fusarium spp.* were rare (*n* = 1; 0.07%).

**Fig. 1:**
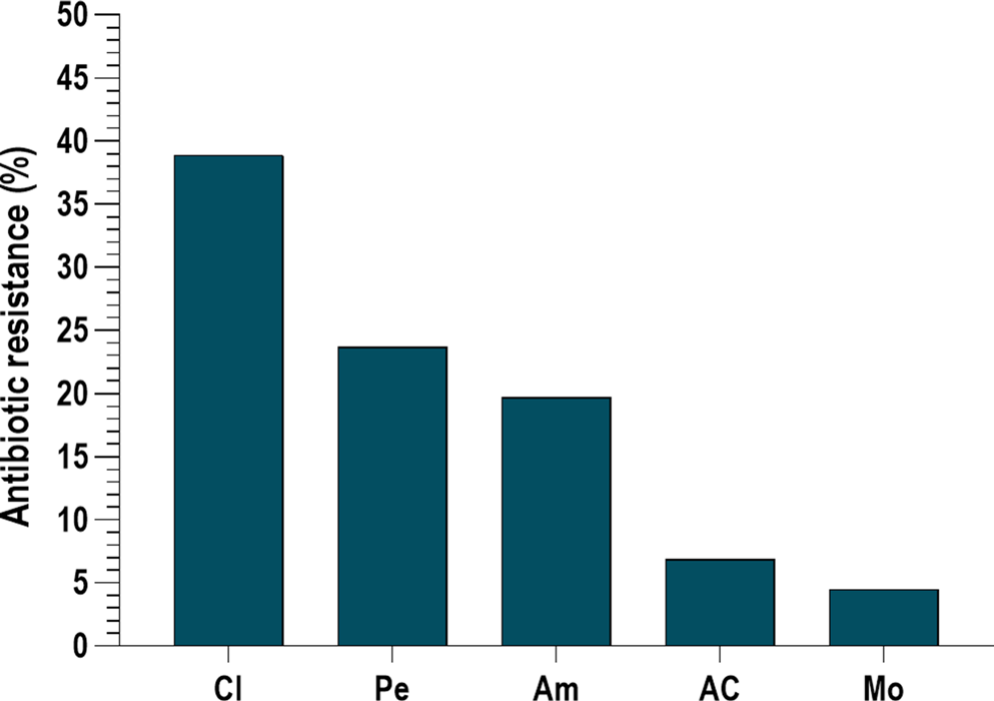
Analysis of pathogen resistance to selected antibiotics at infection level in odontogenic infections. The percentage of infections in which at least one pathogen was resistant to the respective antibiotic is shown. *n =* number of infections with a sensitivity test carried out on the respective antibiotic. **Cl** = Clindamycin (*n =* 380), **Pe** = Penicillin (*n =* 388), **Am** = Amoxicillin (*n =* 411), **AC** = Amoxicillin/clavulanate (*n =* 437), **Mo** = Moxifloxacin (*n =* 221)

An overview of resistance rates identified in the 448 susceptibility tests for antibiotics commonly used in the treatment of odontogenic infections is shown in Fig. 1. The highest resistance rate was observed for clindamycin (38.9%), followed by penicillin (23.7%) and amoxicillin (19.7%). Lower resistance rates were noted for amoxicillin/clavulanate (6.9%) and moxifloxacin (4.5%).

No statistically significant changes in annual resistance rates were observed over the study period for any of the antibiotics analyzed. The results of the corresponding model calculations are presented in Supplementary Table 7.

Additionally, the impact of a reported penicillin hypersensitivity on antibiotic resistance was examined using binary logistic regression. As shown in Table 3, a significantly increased risk of resistance to clindamycin was observed in patients with a documented penicillin hypersensitivity (OR: 2.156; 95%-CI: 1.038–4.480; *p =* 0.04). In this group, resistance rates were 56.3%.

**Table 3:** Results of the binary-logistic regression analysis on the influence of an anamnestically documented penicillin hypersensitivity on the occurrence of resistance to selected antibiotics in odontogenic infections

Antibiotic	OR	95%-CI	*p*-Value
Clindamycin ^a^	2.156	1.038–4.480	**0.040**
Penicillin ^b^	1.496	0.656–3.411	0.338
Amoxicillin ^c^	1.594	0.710–3.575	0.258
Amoxicillin/clavulanate ^d^	0.898	0.204–3.951	0.886
Moxifloxacin ^e^	1.191	0.143–9.942	0.872

The odds ratios (OR) with associated 95% confidence intervals (95%-CI) and p-values for the occurrence of resistance to the respective antibiotic in odontogenic infections treated as inpatients by means of surgical therapy depending on the presence of a reported penicillin hypersensitivity are shown. An infection was considered resistant if at least one pathogen detected was resistant to the respective antibiotic (*n* = number of infections with susceptibility test to the respective antibiotic)

^a^ *n =* 380; ^b^ *n =* 388; ^c^ *n =* 411; ^d^ *n =* 437; ^e^ *n =* 221

Furthermore, the effect of antibiotic resistance on the occurrence of systemic complications and prolonged hospitalization was analyzed using binary logistic regression. A significant association was found between resistance to moxifloxacin and an increased risk of systemic complications. Specifically, the risk of systemic complications was increased by a factor of 10.875 in infections resistant to moxifloxacin (OR: 10.875; 95%-CI: 2.364–50.017; *p =* 0.002). The results of this analysis are presented in Table 4.

**Table 4:** Results of the binary-logistic regression analysis on the influence of microbiological resistance to selected antibiotics on the occurrence of systemic complications in odontogenic infections

Antibiotic	OR	95%-CI	*p*-Value
Clindamycin ^a^	0.658	0.247–1.752	0.402
Penicillin ^b^	0.734	0.204–2.633	0.635
Amoxicillin ^c^	0.708	0.202–2.477	0.589
Amoxicillin/clavulanate ^d^	0.704	0.091–5.448	0.737
Moxifloxacin ^e^	10.875	2.364–50.017	**0.002**

The odds ratios (OR) with associated 95% confidence intervals (95%-CI) and p-values for the occurrence of systemic complications in odontogenic infections treated as inpatients by means of surgical therapy depending on the presence of a resistant infection to the respective antibiotic are shown. An infection was considered resistant if at least one pathogen detected was resistant to the respective antibiotic (*n* = number of infections with susceptibility test to the respective antibiotic)

^a^ *n =* 380; ^b^ *n =* 388; ^c^ *n =* 411; ^d^ *n =* 437; ^e^ *n =* 221

Analysis of the association between antibiotic resistance and prolonged hospitalization revealed no statistically significant correlations for any of the antibiotics investigated. Results are shown in Supplementary Table 8.

## Discussion

As antimicrobial resistance represents one of the most significant global health challenges [32, 41], the role of dentistry as a major source of antibiotic prescriptions has received increasing attention [42]. Therefore, the present study aimed to examine current resistance patterns in odontogenic infections and assess their potential impact on clinically relevant outcomes to guide empiric treatment strategies.

In our cohort, the adjusted mean hospitalization was 6.2 days, closely matching the 5.9 days reported by Heim et al. for a German tertiary care center [43]. International comparisons, however, reveal substantial variability, ranging from approximately 2–3 days in Brazil and the United Kingdom [2, 44] to 9 days in China [11] and 12 days in South Korea [45], which may be influenced by factors such as hospital treatment costs and accessibility of medical facilities [46]. Given this variability, prolonged hospitalization was defined as one standard deviation above the mean to improve international comparability and ensure a clinically meaningful distinction between typical and extended treatment durations.

The incidence and pattern of systemic complications in our cohort were mostly consistent with previously reported rates of 5% to 27% [28, 46, 47], although our incidence was lower at 4.6%. Airway obstructions remained the most frequent and clinically critical complication, occurring in over 50% of systemic cases, consistent with previous reports [1, 18, 48]. Other severe complications, such as sepsis, pneumonia, and descending mediastinitis, have been similarly well documented in the literature [16, 20, 21, 27].

Within this clinical framework, our study identified a broad microbial spectrum comprising 116 bacterial species from 39 genera and six fungal species from three genera. Viridans streptococci, coagulase-negative staphylococci, and *Haemophilus spp.* were the most frequent aerobes, whereas *Prevotella spp.*, *Peptostreptococcus spp.*, and *Veillonella spp.* predominated among strict anaerobes. Overall, aerobic organisms slightly outnumbered anaerobes (55.6% vs. 44.4%), with polymicrobial infections prevailing (average of 2.5 species per infection). *Candida spp.* were the most frequently isolated fungi (2.6%). These findings align well with existing literature highlighting the polymicrobial flora in odontogenic infections [10, 20, 23]. Studies by Grillo et al. [1] and Meinen et al. [10] similarly emphasize viridans streptococci and *Prevotella spp.* as predominant organisms. In contrast, Böttger et al. used 16 S rRNA sequencing to identify *Fusobacterium spp.* and *Porphyromonas spp.* as the main pathogens, suggesting that differences in microbial identification methods can significantly influence the detected microbial spectrum [7]. The slight aerobic predominance observed in our findings contrasts with reports of anaerobic dominance in other studies [1, 7, 29, 36], likely attributable to variations in sampling methodologies [4, 37].

Analysis of 448 susceptibility tests showed the highest resistance to clindamycin (38.9%), followed by penicillin (23.7%) and amoxicillin (19.7%). In contrast, resistance was lower for amoxicillin/clavulanate (6.9%) and moxifloxacin (4.5%). These results align with similar analyses from Neckel et al. [40] and Heim et al. [12], who reported comparable resistance rates at infection level, particularly high rates of clindamycin resistance, in smaller cohort studies.

Despite increasing evidence of high clindamycin resistance in odontogenic infections, it remains frequently prescribed [8, 42, 49]. Our results, however, strongly suggest that clindamycin use should be critically reevaluated, particularly considering its limited suitability as first-line empirical therapy. Conversely, aminopenicillins combined with beta-lactamase inhibitors and moxifloxacin exhibit favorable resistance profiles. Moxifloxacin’s low resistance rate (4.5%) is particularly noteworthy, suggesting its potential as a reliable alternative in penicillin-hypersensitive patients. However, the treatment requires balancing its effectiveness with the possibility of severe adverse effects, including tendinitis and peripheral neuropathy and cardiac complications [50].

The prevalence of resistance to commonly prescribed antibiotics remained stable throughout the twelve-year study period, consistent with findings from Meinen et al. who found no significant increase in aminopenicillin or fluoroquinolone resistance from 2012 to 2019 [10]. However, this stability may partially result from broader microbiological testing over time, including milder infections with potentially more antibiotic-sensitive organisms. Hence, subtle resistance shifts at the species level might have been overlooked, despite their clinical relevance.

Interestingly, a significant association between penicillin hypersensitivity and increased clindamycin resistance was observed, consistent with previous findings [34, 35], suggesting potential selection pressure due to repeated clindamycin use. However, clindamycin resistance was not significantly linked to adverse clinical outcomes. In contrast, moxifloxacin resistance was significantly associated with systemic complications, possibly indicating more virulent or highly resistant pathogens. Resistance to other antibiotics did not significantly affect the risk of prolonged hospitalization or complication rates. The effectiveness of antibiotic switch strategies combined with the low virulence of most resistant strains may explain this observation.

These results reflect ongoing debates in the literature on the clinical relevance of antibiotic resistance. Whereas Kuriyama et al. found resistance to be clinically insignificant [38], others correlated resistant pathogens with more severe disease courses or prolonged hospitalizations [39, 40]. This discrepancy underscores the complexity and multifactorial nature of odontogenic infections, emphasizing that early and adequate surgical intervention is essential to prevent potential negative effects of antibiotic resistance [13, 30, 34].

A key strength of this study is the large, well-defined inpatient cohort and the comprehensive, long-term microbiological assessment, which together provide valuable insights into temporal trends and resistance patterns relevant to clinical practice. The analysis of resistance at the infection level rather than individual isolates enhances clinical applicability, reflecting clinical decision-making in polymicrobial infections more accurately. By linking microbial resistance to clinical outcomes, such as systemic complications and hospitalization duration, the study gains a clinical focus absent from strictly microbiological studies.

Nonetheless, several limitations must be acknowledged. As with all retrospective designs, data completeness and consistency depend on existing documentation, introducing potential bias through missing information and variability in sampling and diagnostic procedures. The exclusive inclusion of inpatient cases undergoing surgical management limits generalizability to milder or conservatively managed infections. Additionally, standard microbiological techniques may overlook uncultivable oral bacteria and lack consistent species-level differentiation, potentially underestimating microbial diversity and resistance patterns. Moreover, not all isolates underwent uniform susceptibility testing, leading to variable sample sizes across antibiotics. The small number of systemic complications and the reduced number of cases with complete susceptibility data further constrained the feasibility of multivariable analyses incorporating additional clinical risk factors [51].

Despite these limitations, the findings underscore the need for structured antibiotic stewardship initiatives in dentistry and support adherence to guideline-concordant prescribing practices. The high rate of clindamycin resistance, especially among penicillin-hypersensitive patients, raises important concerns about its continued use as a first-line alternative. Given the observed efficacy and low resistance rates, amoxicillin/clavulanate and moxifloxacin should be prioritized as empirical agents, particularly in severe cases, or in the case of penicillin hypersensitivity, the latter should be preferred. Additionally, standardizing and verifying hypersensitivity documentation together with systematic delabelling procedures would decrease inappropriate antibiotic use. Therefore, continuous education, practice audits, and adherence to guidelines on odontogenic infections could further promote rational antibiotic prescribing, addressing persistent disparities in antibiotic use internationally.

Future research should prioritize prospective studies using standardized microbiological methods to validate these findings and increase generalizability. Molecular approaches, such as 16 S rRNA sequencing or next-generation sequencing, could enable more comprehensive characterization of oral microbial communities, including uncultivable species, and deepen the understanding of pathogenic mechanisms and resistance dynamics. Prospective studies using standardized microbiological diagnostics and antimicrobial susceptibility testing, together with multivariable analyses incorporating relevant clinical risk factors, are needed to clarify the independent impact of antimicrobial resistance on patient outcomes. Such evidence would support improved risk stratification and guide more targeted therapeutic decisions. In addition, randomized controlled trials comparing empirical treatment regimens may further contribute to optimizing the clinical management of odontogenic infections. In conclusion, this comprehensive retrospective analysis provides critical insights into antibiotic resistance patterns in odontogenic infections. Persistent high resistance to clindamycin and its implications, particularly in penicillin-hypersensitive patients, emphasizes the need for careful antibiotic stewardship and re-evaluation of current treatment guidelines. Moxifloxacin provides a reassuring alternative for empirical therapy in patients with penicillin hypersensitivity, while resistance to moxifloxacin may indicate highly resistant infections associated with an increased risk of systemic complications. Strengthening microbiological diagnostic practices and hypersensitivity verification processes will be instrumental in improving patient outcomes.

## Supplementary Information

Below is the link to the electronic supplementary material.

Supplementary Material 1 (DOCX 126 KB)

## Data Availability

The datasets used and/or analyzed during the current study are available from the corresponding author on reasonable request.
