# Supplementary material for: Clinical impact of antibiotic resistance in odontogenic infections: a 12-year analysis of 740 cases
Source: Clin Oral Investig. 2025 Dec 3;29(12):597. doi: 10.1007/s00784-025-06687-6 (PMC12675659; doi:10.1007/s00784-025-06687-6)
Supplement: Supplementary file 1 — Supplementary Material 1 (DOCX 126 KB) [file 784_2025_6687_MOESM1_ESM.docx]

**SUPPLEMENTARY FIGURES AND TABLES**


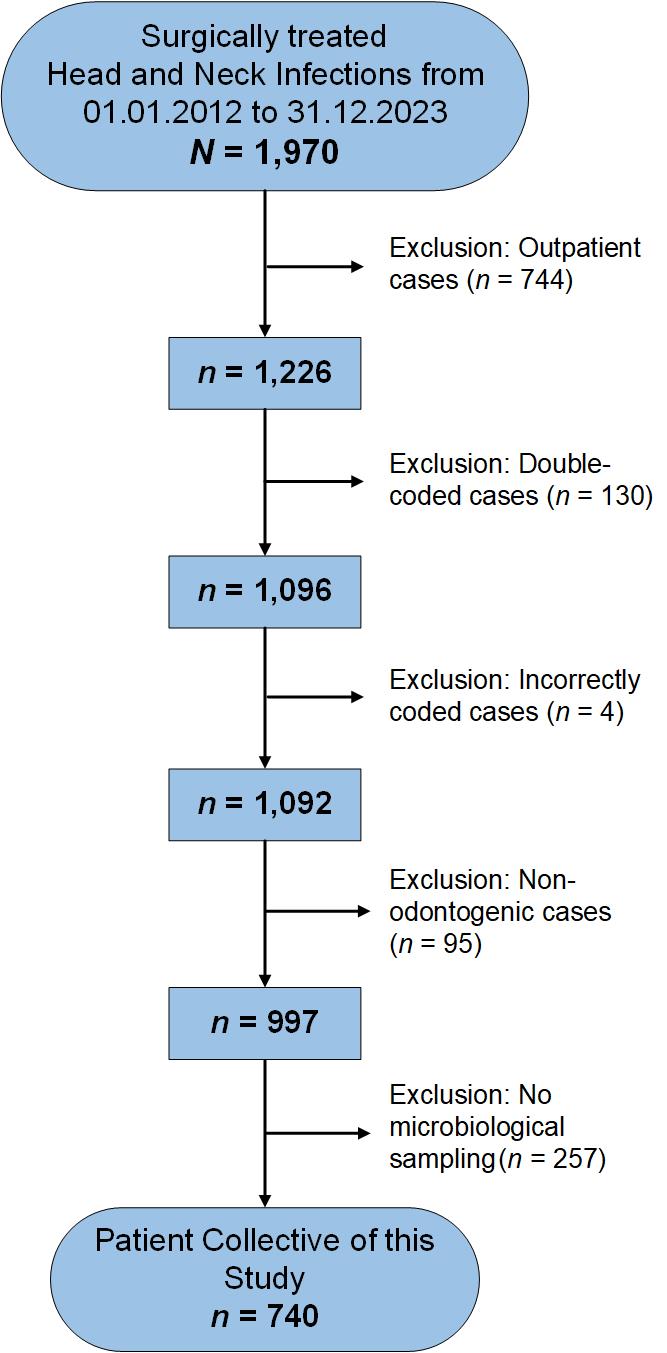


***Supplementary Figure 1*:** **Exclusion criteria of the study population**

***Supplementary Table 1*: ICD-10-GM codes used for case selection**

| ICD-10-GM code | Description |
| --- | --- |
| H05.0 | Acute inflammation of orbit |
| J34.0 | Abscess, furuncle and carbuncle of nose |
| J36 | Peritonsillar abscess |
| J39.0 | Retropharyngeal and parapharyngeal abscess |
| J39.1 | Other abscess of pharynx |
| K00.7 | Teething syndrome |
| K04.0 – K04.9 | Diseases of pulp and periapical tissues |
| K10.20 – K10.29 | Inflammatory conditions of jaws |
| K10.3 | Alveolitis of jaws |
| K11.3 | Abscess of salivary gland |
| K12.20 – K12.29 | Cellulitis and abscess of mouth |
| L02.0 | Cutaneous abscess, furuncle and carbuncle of face |

***Supplementary Table 2*: OPS** **codes used for case selection**

| OPS code | Description |
| --- | --- |
| **5-240** | **Operations on gums, alveolae and jaw: Incision into the gum and osteotomy of the alveolar ridge** |
| 5-240.0 | Incision into the gum |
| 5-240.1 | Periodontal drainage |
| **5-270** | **Other operations on mouth and face: External incision and drainage in mouth, jaw and face area** |
| 5-270.0 | Temporal |
| 5-270.1 | Periorbital |
| 5-270.2 | Paranasal |
| 5-270.3 | Cheek area |
| 5-270.4 | Parotid region |
| 5-270.5 | Submandibular |
| 5-270.6 | Submandibular, mandibular angle area |
| 5-270.7 | Submental |
| 5-270.8 | Tongue base |
| 5-270.9 | Sternocleidomastoid muscle area |
| 5-270.x | Other |
| 5-270.y | Unspecified |
| **5-273** | **Other operations on mouth and face: Incision, excision and destruction in the oral cavity** |
| 5-273.0 | Incision and drainage, vestibular submucosal |
| 5-273.1 | Incision and drainage, vestibular subperiosteal |
| 5-273.2 | Incision and drainage, sublingual |
| **5-280** | **Operations in the nasopharyngeal and oropharyngeal area: Transoral incision and drainage of a pharyngeal or parapharyngeal abscess** |
| 5-280.0 | (Peri)tonsillar |
| 5-280.1 | Parapharyngeal |
| 5-280.2 | Retropharyngeal |
| 5-280.3 | In the floor of mouth area |
| 5-280.x | Other |
| 5-280.y | Unspecified |

***Supplementary Table 3*: Distribution of comorbidities and patient-related risk factors**

Presentation of relevant comorbidities and patient-related risk factors during the observation period from 2012 to 2023 for inpatients with odontogenic infections. The absolute numbers and percentages relative to the total study population (*n* = 740, unless otherwise specified) are shown.

| Risk factor | Number of cases | Percent (%) |
| --- | --- | --- |
| Cardiovascular disease | 311 | 42.0 |
| Diabetes mellitus | 110 | 14.9 |
| COPD | 45 | 6.1 |
| OSAS | 11 | 1.5 |
| Severe renal impairment | 12 | 1.6 |
| Liver disease | 10 | 1.4 |
| Immunosuppression | 60 | 8.1 |
| Neurological disease | 87 | 11.8 |
| Psychiatric disorders | 95 | 12.8 |
| Oral anticoagulation | 184 | 24.9 |
| Chronic alcohol addiction | 49 | 6.6 |
| Chronic tobacco addiction ^b^ | 312 | 47.2 |
| Penicillin allergy | 60 | 8.1 |

^b^ *n =* 661 (available tobacco addiction data)

***Supplementary Table 4*: Distribution of the maxillofacial spaces involved**

Presentation of the maxillofacial spaces involved during the observation period from 2012 to 2023 for inpatients with odontogenic infections. The absolute number of spaces involved and the percentage distribution in relation to the total number of all maxillofacial spaces involved in the study (*n* = 659) are shown. Several maxillofacial spaces could be affected simultaneously in the same patient.

| Maxillofacial Space | Number of spaces involved | Percent (%) |
| --- | --- | --- |
| Peri-/Submandibular | 369 | 56.0 |
| Buccal | 104 | 15.8 |
| Canine fossa | 59 | 9.0 |
| Mental/Submental | 37 | 5.6 |
| Masseterikomandibular | 23 | 3.5 |
| Sublingual | 22 | 3.3 |
| Para-/Retropharyngeal | 21 | 3.2 |
| Pterygomandibular | 8 | 1.2 |
| Retromaxillary | 7 | 1.1 |
| Temporal | 5 | 0.8 |
| Parotid space | 2 | 0.3 |
| Orbital | 1 | 0.2 |
| Para-/Peritonsilliar | 1 | 0.2 |

***Supplementary Table 5*: Distribution of systemic complications resulting from odontogenic infections**

Presentation of the systemic complications that occurred during the observation period from 2012 to 2023 for inpatients with odontogenic infections. The absolute case numbers and the percentage distribution in relation to the total number of patients included in the analysis (*n* = 740) are shown. Several complications could occur simultaneously in the same patient.

| Complication | Number of cases | Percent (%) |
| --- | --- | --- |
| Airway obstruction | 20 | 2.7 |
| Sepsis | 17 | 2.3 |
| Pneumonia | 9 | 1.2 |
| Septic shock | 7 | 0.9 |
| Mediastinitis | 5 | 0.7 |
| Critical illness polyneuropathy | 3 | 0.4 |
| Disseminated intravascular coagulopathy | 2 | 0.3 |
| Brain abscess | 1 | 0.1 |
| Diabetic ketoacidosis | 1 | 0.1 |

***Supplementary Table 6:* Frequency of detected microorganisms**

Presentation of the detected microorganisms from microbiological reports. Absolute frequencies and percentage distribution in relation to the total of all isolates (*n =* 1,516) are shown.

| Pathogens | Number of isolates | Percent (%) |
| --- | --- | --- |
| Viridans streptococci | 435 | 28,7 |
| *Prevotella spp.* | 337 | 22,2 |
| Coagulase-negative staphylococci | 105 | 6,9 |
| Peptostreptococci | 71 | 4,7 |
| *Veillonella spp.* | 71 | 4,7 |
| *Fusobacterium spp.* | 68 | 4,5 |
| *Haemophilus spp.* | 54 | 3,6 |
| *Bacteroides spp.* | 50 | 3,3 |
| Propionibacteria | 43 | 2,8 |
| *Candida spp.* | 39 | 2,6 |
| *Neisseria spp.* | 36 | 2,4 |
| β-hemolytic streptococci | 33 | 2,2 |
| *Eikenella corrodens* | 31 | 2,0 |
| Peptococci | 25 | 1,6 |
| *Staphylococcus aureus* | 20 | 1,3 |
| *Capnocytophaga spp.* | 14 | 0,9 |
| *Escherichia coli* | 12 | 0,8 |
| Enterococci | 10 | 0,7 |
| *Klebsiella spp.* | 9 | 0,6 |
| *Actinomyces spp.* | 5 | 0,3 |
| *Enterobacter cloacae complex* | 5 | 0,3 |
| *Pseudomonas aeruginosa* | 5 | 0,3 |
| *Bifidobacterium spp.* | 4 | 0,3 |
| *Cutibacterium acnes* | 4 | 0,3 |
| *Corynebacterium spp.* | 3 | 0,2 |
| *Morganella morganii* | 3 | 0,2 |
| *Serratia marcescens* | 3 | 0,2 |
| *Slackia exigua* | 3 | 0,2 |
| *Citrobacter spp.* | 2 | 0,1 |
| *Porphyromonas spp.* | 2 | 0,1 |
| *Proteus mirabilis* | 2 | 0,1 |
| *Acinetobacter baumannii complex* | 1 | 0,1 |
| *Aggregatibacter spp.* | 1 | 0,1 |
| *Bacillus spp.* | 1 | 0,1 |
| *Campylobacter rectus* | 1 | 0,1 |
| *Gemella sp.* | 1 | 0,1 |
| *Lactococcus spp.* | 1 | 0,1 |
| *Leuconostoc spp.* | 1 | 0,1 |
| *Parvimonas micra* | 1 | 0,1 |
| *Pasteurella spp.* | 1 | 0,1 |
| *Pediococcus pentosaceus* | 1 | 0,1 |
| *Rothia dentocariosa* | 1 | 0,1 |
| *Fusarium spp.* | 1 | 0,1 |

***Supplementary Table 7*: Results of the Poisson regression to analyze the annual development of resistance to selected antibiotics in the period from 2012 to 2023.**

The incidence rate ratios (IRR), 95% confidence intervals (95%-CI) and p-values for temporal trends in resistance rates at infection level in the observation period are shown. An infection was considered resistant if at least one pathogen detected was resistant to the respective antibiotic. The regression analyses were performed using the annual number of susceptibility tests per antibiotic as an offset.

| Antibiotic | IRR | 95%-CI | *p-*Value |
| --- | --- | --- | --- |
| Clindamycin | 1.015 | 0.969 – 1.064 | 0.521 |
| Penicillin | 0.974 | 0.919 – 1.031 | 0.363 |
| Amoxicillin | 0.985 | 0.926 – 1.049 | 0.645 |
| Amoxicillin/clavulanate | 1.071 | 0.955 – 1.202 | 0.241 |
| Moxifloxacin | 1.118 | 0.934 – 1.339 | 0.225 |

Offset: Annual number of susceptibility tests per antibiotic

***Supplementary Table 8*: Results of the binary-logistic regression analysis on the influence of microbiological resistance to selected antibiotics on prolonged hospitalization in hospitalized odontogenic infections**

The odds ratios (OR) with associated 95% confidence intervals (95%-CI) and p-values for prolonged hospitalization in odontogenic infections treated as inpatients by means of surgical therapy depending on the presence of a resistant infection to the respective antibiotic are shown. An infection was considered resistant if at least one pathogen detected was resistant to the respective antibiotic (*n* = number of infections with susceptibility test to the respective antibiotic).

| Antibiotic | OR | 95%-CI | *p-*Value |
| --- | --- | --- | --- |
| Clindamycin ^a^ | 1.370 | 0.678 – 2.769 | 0.380 |
| Penicillin ^b^ | 1.674 | 0.778 – 3.601 | 0.187 |
| Amoxicillin ^c^ | 1.561 | 0.721 – 3.382 | 0.259 |
| Amoxicillin/clavulanate ^d^ | 1.791 | 0.583 – 5.503 | 0.309 |
| Moxifloxacin ^e^ | 0.950 | 0.114 – 7.893 | 0.962 |

^a^ *n* = 380; ^b^ *n* = 388; ^c^ *n* = 411; ^d^ *n* = 437; ^e^ *n* = 221
